# Uremic Toxins Enhance Statin-Induced Cytotoxicity in Differentiated Human Rhabdomyosarcoma Cells

**DOI:** 10.3390/toxins6092612

**Published:** 2014-09-03

**Authors:** Hitoshi Uchiyama, Masayuki Tsujimoto, Tadakazu Shinmoto, Hitomi Ogino, Tomoko Oda, Takuya Yoshida, Taku Furukubo, Satoshi Izumi, Tomoyuki Yamakawa, Hidehisa Tachiki, Tetsuya Minegaki, Kohshi Nishiguchi

**Affiliations:** 1 Pharmacovigilance and Post Marketing Surveillance Department, Towa Pharmaceutical Co. Ltd., 2-11, Shinbashi-cho, Kadoma, Osaka 571-8580, Japan; 2 Department of Clinical Pharmacy, Faculty of Pharmaceutical Science, Kyoto Pharmaceutical University, 5 Misasagi nakauchi-cho, Yamashina-ku, Kyoto 607-8414, Japan; 3 Department of Pharmacy Service, Shirasagi Hospital, 7-11-23 Kumata, Higashisumiyoshi-ku, Osaka 546-0002, Japan; 4 Department of Medicine, Shirasagi Hospital, 7-11-23 Kumata, Higashisumiyoshi-ku, Osaka 546-0002, Japan; 5 Research and Development Division, Towa Pharmaceutical Co. Ltd., Kyoto Research Park KISTIC#202, 134, Chudoji Minami-Machi, Shimogyo-ku, Kyoto 600-8813, Japan

**Keywords:** cytotoxicity, statin, uremic toxin, end-stage renal failure, rhabdomyolysis

## Abstract

The risk of myopathy and rhabdomyolysis is considerably increased in statin users with end-stage renal failure (ESRF). Uremic toxins, which accumulate in patients with ESRF, exert cytotoxic effects that are mediated by various mechanisms. Therefore, accumulation of uremic toxins might increase statin-induced cytotoxicity. The purpose of this study was to determine the effect of four uremic toxins—hippuric acid, 3-carboxy-4-methyl-5-propyl-2-furanpropionate, indole-3-acetic acid, and 3-indoxyl sulfate—on statin-induced myopathy. Differentiated rhabdomyosarcoma cells were pre-treated with the uremic toxins for seven days, and then the cells were treated with pravastatin or simvastatin. Cell viability and apoptosis were assessed by viability assays and flow cytometry. Pre-treatment with uremic toxins increased statin- but not cisplatin-induced cytotoxicity (*p* < 0.05 *vs.* untreated). In addition, the pre-treatment increased statin-induced apoptosis, which is one of the cytotoxic factors (*p* < 0.05 *vs.* untreated). However, mevalonate, farnesol, and geranylgeraniol reversed the effects of uremic toxins and lowered statin-induced cytotoxicity (*p* < 0.05 *vs.* untreated). These results demonstrate that uremic toxins enhance statin-induced apoptosis and cytotoxicity. The mechanism underlying this effect might be associated with small G-protein geranylgeranylation. In conclusion, the increased severity of statin-induced rhabdomyolysis in patients with ESRF is likely due to the accumulation of uremic toxins.

## 1. Introduction

3-Hydroxy-3-methylglutaryl coenzyme A (HMG-CoA) reductase inhibitors (statins), which are potent inhibitors of cholesterol biosynthesis and mevalonate reduction, are widely used for prevention of atherosclerotic cardiovascular disease due to hypercholesterolemia [1,2,3]. However, statins induce serious adverse drug reactions (ADRs) such as myopathies and rhabdomyolysis. Rhabdomyolysis increases the levels of serum creatine kinase, urine and serum myoglobin, and presents clinically as numbness and paralysis of all four limbs. One of the risk factors for rhabdomyolysis is renal dysfunction [4]. The plasma concentrations of atorvastatin, pravastatin (PRV), and lovastatin become significantly elevated in patients with end-stage renal failure (ESRF)* versus* those with normal renal function [5,6,7,8]. Hermann *et al.* reported that the plasma concentrations of atorvastatin increase several-fold in patients with atorvastatin-induced myopathy [9]. It is possible that one of the risk factors for statin-induced rhabdomyolysis in patients with ESRF is elevated plasma concentrations of statin; however, the risk cannot be explained by this alone. For example, the 521TC genotype in the organic anion transporting polypeptide (OATP) 1B1, an uptake transporter located at the sinusoidal membrane of human hepatocytes, affects the plasma concentrations of atorvastatin, simvastatin (SIM), and PRV [10,11,12]. This genotype is associated with an elevated risk of SIM-induced ADRs, but not with the risk of atorvastatin or PRV-induced ADRs [13]. Therefore, it is possible that there are the other specific risk factors for statin-induced myopathy in patients with ESRF.

Several uremic toxins are present at pathological concentrations in patients with ESRF as a consequence of renal dysfunction. The uremic toxins such as indole-3-acetic acid, 3-indoxyl sulfate, and 3-carboxy-4-methyl-5-propyl-2-furanpropionate (CMPF) promote cytotoxicity by inducing oxidative stress in rat neutrophils, rat lymphocytes, human aortic smooth muscle cells, and human proximal tubular cells, respectively [14,15,16]. Furthermore, hippuric acid might cause muscular weakness by inhibiting glucose utilization in an *in vitro* model of renal dysfunction [17]. It is possible that uremic toxins are risk factors for statin-induced myopathy, although this has not yet been confirmed. Statins also inhibit HMG-CoA reductase, which catalyzes the rate-limiting step in the mevalonate pathway, thus reducing the concentrations of cholesterol and the biosynthesis of mevalonate pathway-related compounds. For example, statins reduce biosynthesis of farnesyl pyrophosphate (FPP) and geranylgeranyl pyrophosphate (GGPP). Subsequently, statins decrease activity of small G proteins such as Ras and Rho, which are associated with apoptotic signals [18]. Reduced biosynthesis of coenzymeQ-10 (CoQ_10_) results in abnormal oxidative phosphorylation in the mitochondrial electron transport chain, which damages cellular homeostasis [19]. Furthermore, interleukin-(IL)-1β- and IL-6-induced oxidative stress promotes hepatic cholesterol synthesis [20]. Therefore, we hypothesized that uremic toxins affect the biosynthesis of mevalonate pathway-related compounds, thus augmenting statin-induced cytotoxicity in striated muscle.

This aim of this study was to determine whether uremic toxins affect statin-induced cytotoxicity, and investigate the role of the mevalonate pathway in augmenting statin-induced cytotoxicity caused by uremic toxins in human rhabdomyosarcoma (RD) cells.

## 2. Results and Discussion

### 2.1. Effects of Pre-Treatment with Uremic Toxin (CMPF, Hippuric Acid, Indole-3-Acetic Acid, or 3-Indolxyl Sulfate) on Statin-Induced Cytotoxicity in Differentiated RD Cells

**Figure 1:**
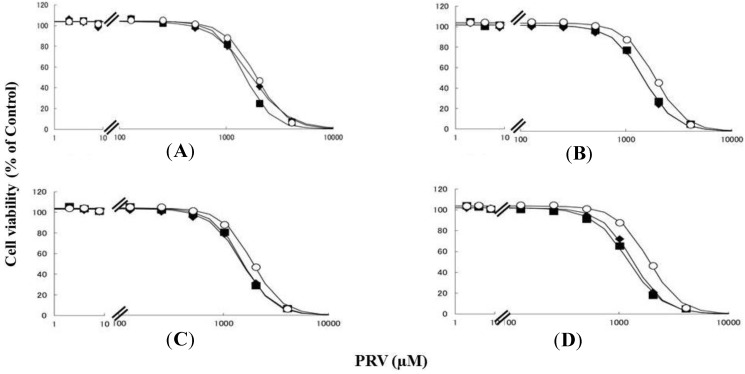
RD cells were seeded at a density of 5 × 10^3^ cells/well into 96-well plates. After three days, the media were changed to differentiation medium containing uremic toxins at concentrations equivalent to total or unbound serum concentrations. After another seven days, cells were incubated with differentiation medium containing different concentrations of PRV for three days. The cytotoxicity of PRV was determined using CellQuanti-Blue™ Cell Viability Assay Kits. Data are presented as mean ± S.D. (*n* = 3). *Open circles*, untreated cells; *closed rhombus*, cells pre-treated with uremic toxins at concentrations equivalent to unbound serum levels in ESRF patients; *closed squares*, cells pre-treated with uremic toxins at concentrations equivalent to total serum levels in ESRF patients. (**A**) CMPF; (**B**) Hippuric acid; (**C**) Indole-3-acetic acid; (**D**) 3-Indoxyl sulfate.

**Figure 2:**
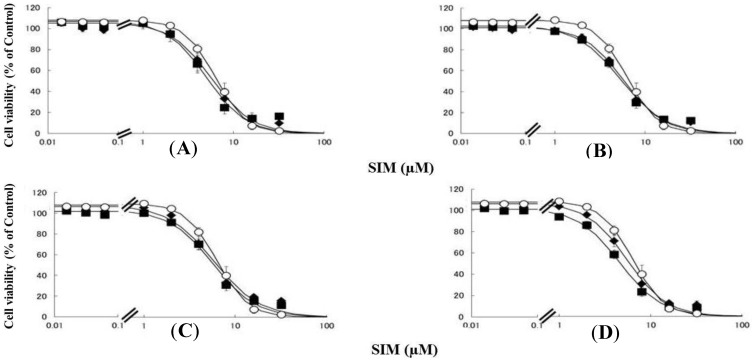
RD cells were seeded at a density of 5 × 10^3^ cells/well in 96-well plates. After three days, the media were changed to differentiation medium containing uremic toxins at concentrations equivalent to total or unbound serum levels. After seven days, the cells were incubated with differentiation medium containing various concentrations of SIM for a further three days. The cytotoxicity of SIM was then determined using CellQuanti-Blue™ Cell Viability Assay Kits. Data are presented as mean ± S.D. (*n* = 3). *Open circles*, untreated cells; *closed rhombus*, cells pre-treated with uremic toxins at concentrations equivalent to unbound serum levels in ESRF patients; *closed squares*, cells pre-treated with uremic toxins at concentrations equivalent to total serum levels in ESRF patients. (**A**) CMPF; (**B**) Hippuric acid; (**C**) Indole-3-acetic acid; (**D**) 3-Indoxyl sulfate.

Rhabdomyolysis is an ADR associated with statin use, which develops in renal dysfunction. It might be caused by the increased sensitivity of myocytes to statins. This study demonstrated that pre-treating RD cells with CMPF, hippuric acid, indole-3-acetic acid, or 3-indoxyl sulfate shifted the survival curves in response to PRV (Figure 1) and SIM (Figure 2) to the left. Each LC_50_ value except 180 µM hippuric acid, or 3 µM indole-3-acetic acid with SIM also decreased significantly (Table 1). This augmentation of statin-induced cytotoxicity is caused by elevated serum concentrations of CMPF, hippuric acid, indole-3-acetic acid, or 3-indoxyl sulfate. In this study, the range of PRV and SIM levels is higher than in the *in vivo* situation, e.g., Cmax 48.1 ng/mL (108 nM) for PRV in hemodialysis patients [5], and Cmax 8.9–13.0 ng/mL (21–31 nM) and 2.2–6.6 ng/mL (5–15 nM) in healthy caucasians for SIM and SIM acid [11]. In addition, Kobayashi *et al.* reported ranges of PRV and SIM levels to measure caspase-3/7 activity ratio between 0.1 and 100 μM [21]. Thus, higher concentration of statins is needed for inducing muscle disorder *in vitro* and statin users with myopathy or rhabdomyolysis might have a higher statin concentration in the muscle. Therefore, the reason why the current range of PRV and SIM levels was chosen in the present study is to assess clearly effects of uremic toxins on cells. It is of note that, myopathy and rhabdomyolysis do not develop in all statin users and more often in CKD patients [4]. Our results suggest that the accumulation of uremic toxins enhances statin-induced myopathy and rhabdomyolysis in patients with ESRF.

In this study, the viability, cell proliferation, the accumulation of statins, and cytomorphology of differentiated RD cells were unaffected by pre-treated with CMPF, hippuric acid, indole-3-acetic acid, or 3-indoxyl sulfate (data not shown). In addition, the simultaneous treatment of differentiated RD cells with uremic toxin (CMPF, hippuric acid, indole-3-acetic acid, or 3-indolxyl sulfate) and statins did not affect statin-induced cytotoxicity (data not shown), unlike pre-treatment. These results suggest that the mechanism by which uremic toxins modulate statin-induced cytotoxicity, involves prolonged exposure of differentiated RD cells to uremic toxins.

**Table 1:** LC_50_ values for statins in differentiated RD cells treated with uremic toxins.

Uremic toxins		LC_50_ Values (95% Confidence Interval)	
		PRV (µM)	SIM (µM)
Untreated		1865 (1819–1919)	6.30 (6.05–6.54)
CMPF	2 µM	1675 (1570–1779) *	5.73 (5.17–6.29) *
	400 µM	1438 (1398–1549) *^,†^	5.05 (4.61–5.50) *
Hippuric acid	180 µM	1463 (1420–1506) *	5.79 (5.46–6.11)
	400 µM	1463 (1419–1506) *	5.37 (5.06–5.68) *
Indole-3-acetic acid	3 µM	1484 (1438–1529) *	5.84 (5.40–6.28)
	20 µM	1513 (1464–1583) *	5.70 (5.36–6.04) *
3-indoxyl sulfate	20 µM	1342 (1303–1381) *	5.49 (5.18–5.79) *
	200 µM	1225 (1188–1261) *^,†^	4.57 (4.34–4.80) *^,†^

Notes: SIM, simvastatin; PRV, pravastatin; CMPF, 3-carboxy-4-methyl-5-propyl-2-furanpropionate. * Non-overlapping 95% confidence intervals, *vs.* untreated cells; ^†^ Non-overlapping 95% confidence intervals, *vs.* unbound serum concentrations.

### 2.2. Effects of Pre-treatment with Uremic Toxins on Statin-Induced Cytotoxicity and Apoptosis in Differentiated RD Cells

**Figure 3:**
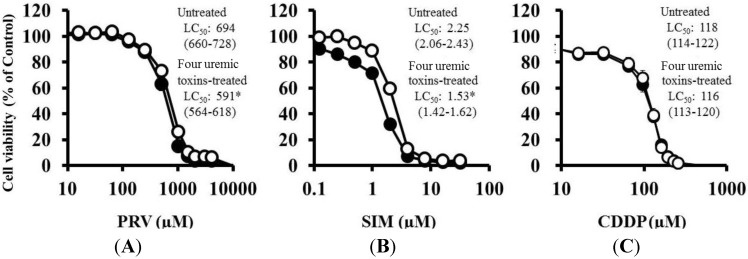
RD cells were seeded at a density of 5 × 10^3^ cells/well into 96-well plates. After three days, the media were changed to the differentiation medium containing four uremic toxins at concentrations equivalent to the unbound serum levels in patients with ESRF. After seven days, the cells were incubated with differentiation medium containing various concentrations of statins or CDDP for three days. The cytotoxicity of statins and CDDP was then determined using CellQuanti-Blue™ Cell Viability Assay Kits. Data are presented as mean ± S.D. (*n* = 3 or 4). *Open circles*, untreated cells; *closed circles*, cells pre-treated with uremic toxins at concentrations equivalent to unbound serum levels in ESRF patients. * Non-overlapping 95% confidence intervals. (**A**) PRV; (**B**) SIM; (**C**) CDDP.

**Figure 4:**
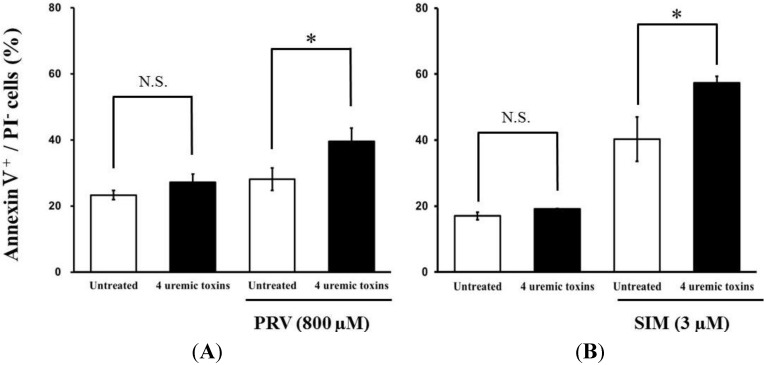
RD cells were seeded at a density of 1.5 × 10^5^ cells/well into 6-well plates. After three days, the media were changed to differentiation medium containing four uremic toxins at concentrations equivalent to the unbound serum levels in patients with ESRF. After seven days, the cells were incubated with differentiation medium containing statins for 36 h. Statin-induced apoptosis was then determined using flow cytometry with Annexin V-FITC/propidium iodide (PI) double staining. Data are presented as mean ± S.D. (*n* = 3 or 4). Significant differences from untreated cells were determined using *post hoc* Tukey-Kramer multiple comparison test (* *p* < 0.05; N.S., not significant). *Open bars*, untreated cells; *closed bars*, cells pre-treated with four uremic toxins at concentrations equivalent to unbound serum levels in ESRF patients. (**A**) PRV; (**B**) SIM.

Pre-treatment with the mixture of four uremic toxins at concentrations equivalent to the unbound serum levels in patients with ESRF also shifted the survival curves to the left in response to both PRV (Figure 3A) and SIM (Figure 3B). Each LC_50_ value was also decreased significantly. In contrast, Cisplatin (CDDP)-induced cytotoxicity was unchanged by pre-treatment with uremic toxins (Figure 3C). Pre-treatment with the mixture of four uremic toxins alone did not affect the number of apoptotic cells; however, it enhanced statin-induced apoptosis (Figure 4). Prolonged exposure to the four uremic toxins increased statin-induced cytotoxicity and apoptosis in differentiated RD cells. However, they did not affect CDDP-induced cytotoxicity, which has a different mechanism of action. CDDP stimulates ATR, p53, p73, and mitogen-activated protein kinase by forming inter- and intra-strand cross-linked DNA adducts, ultimately resulting in apoptosis [22]. These results suggest the tested uremic toxins only increase sensitivity to specific drugs, such as statins.

### 2.3. Effects of Mevalonate Pathway-Related Compounds on Statin-Induced Apoptosis in Differentiated RD Cells Treated with Four Uremic Toxins

**Figure 5:**
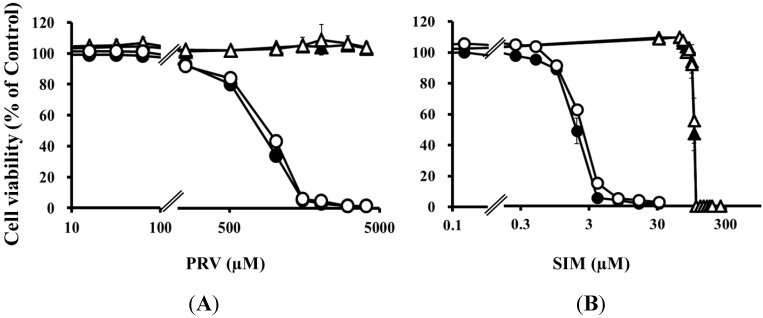
RD cells were seeded at a density of 5 × 10^3^ cells/well into 96-well plates. After three days, the media were changed to differentiation medium containing four uremic toxins at concentrations equivalent to unbound serum levels in ESRF patients. After seven days, the cells were incubated with differentiation medium containing various concentrations of statins in the absence or presence of 1000 µM mevalonate for three days. The cytotoxicity of statins was determined using CellQuanti-Blue™ Cell Viability Assay Kits. Each point represents the mean ± S.D. (*n* = 3 or 4). *Open circles*, untreated cells; *closed circles*, cells treated with four uremic toxins; *open triangles*, cells treated with mevalonate; *closed triangles*, cells treated with four uremic toxins and mevalonate.

Statins function by reducing mevalonate concentrations via HMG-CoA reductase inhibition. Simultaneous treatment with mevalonate markedly reduced statin-induced cytotoxicity, and completely prevented the increased statin-induced cytotoxicity caused by pre-treatment with the mixture of four uremic toxins (Figure 5 and Table 2), suggesting that increased cytotoxicity was associated with inhibition of the mevalonate pathway.

**Table 2:** LC_50_ values for statins with and without mevalonate in differentiated RD cells treated with uremic toxins

	LC_50_ Values (95% Confidence Interval)			
	PRV (µM)	PRV (µM) + Mevalonate (µM)	SIM (µM)	SIM (µM) + Mevalonate (µM)
Untreated	898 (857–940)	N.C.	2.23 (2.15–2.31)	105 (103–106)
Four uremic toxins	814 (786–842) *	N.C.	1.97 (1.89–2.04) *	103 (102–104)

Notes: PRV, pravastatin; SIM, simvastatin. * Non-overlapping 95% confidence intervals, *vs.* untreated cells; N.C., not Calculated.

**Figure 6:**
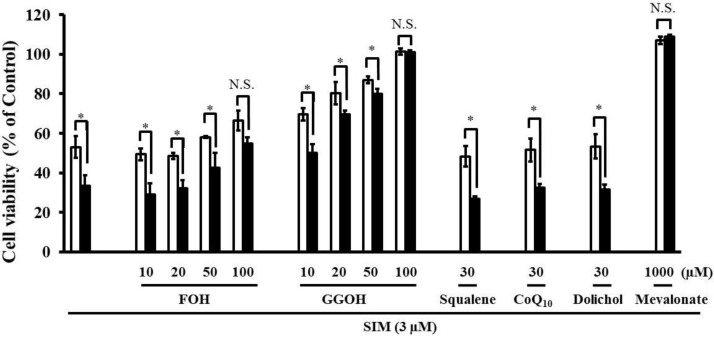
RD cells were seeded at a density of 5 × 10^3^ cells/well into 96-well plates. After three days, the media were changed to differentiation medium containing four uremic toxins at concentrations equivalent to unbound serum levels in ESRF patients. After seven days, the cells were incubated with differentiation medium containing 3 µM SIM in the absence and presence of mevalonate Squalene FOH CoQ_10_, and GGOH for three days. The cytotoxicity of SIM was then determined using CellQuanti-Blue™ Cell Viability Assay Kits. Each point represents the mean ± S.D. (*n* = 3). Significant differences from the untreated control which is no toxin, no statin, no mevalonate pathway-related compound, and same incubation time were determined using unpaired *post hoc* Tukey-Kramer multiple comparison test (* *p* < 0.05; N.S., not significant). *White bars*, untreated cells; *black bars*, cells pre-treated with four uremic toxins at concentrations equivalent to unbound serum levels in ESRF patients.

Simultaneous treatment with farnesol (FOH), or geranylgeraniol (GGOH) caused a dose-dependent suppression of the increased SIM-induced cytotoxicity caused by pre-treatment with the mixture of four uremic toxins (Figure 6). The increase in statin-induced cytotoxicity caused by four uremic toxins was completely abrogated by GGOH, the precursor of GGPP, and weakly suppressed by FOH, the precursor of FPP. In addition, the combination of four uremic toxins potentiated statin-induced apoptosis. FPP is converted into GGPP by GGPP synthase [23]. Subsequently, the reduction of GGPP induces apoptosis via RhoA inactivation in fibroblasts [18]. Therefore, it is possible that the potentiation of statin-induced cytotoxicity and apoptosis by uremic toxins is caused by the suppression of small G-protein geranylgeranylation. Moreover, viability of cells treated with 1000 μM mevalonate and 100 μM GGOH is better compared to control, which may be caused by the beneficial effect of GGOH and mevalonate on cell growth and differentiation. Mevalonate being a precursor in the synthesis of FPP and GGPP, and which being associated with activity of small G proteins such as Ras and Rho, both playing crucial roles in cell growth and differentiation.

In contrast, treatment with squalene, CoQ_10_, and dolichol, which are biosynthetic compounds produced by the mevalonate pathway, did not suppress the increase in statin-induced cytotoxicity caused by four uremic toxins (Figure 6). It has been reported that CoQ_10_ is involved in mitochondrial electron transport and oxidative phosphorylation [19], but that it cannot suppress statin-induced skeletal muscle toxicity [24]. Therefore, these results suggest that mitochondrial electron transport and oxidative phosphorylation and CoQ_10_ are not involved in the acceleration of statin-induced apoptosis and cytotoxicity by uremic toxins on RD cells.

Uremic toxins exert various effects on cells by generating proinflammatory cytokines via the induction of oxidative stress [25]. For example, Zhang *et al.* reported that interleukin-1β increased HMG-CoA reductase mRNA, which caused the intracellular accumulation of LDL-cholesterol in glomerular endothelial cells [26]. In addition, Biswas *et al.* reported that oxidative stress altered many proteins function and the affinity for protein substrates by oxidizing cysteine residues [27]. These reports suggested that uremic toxins might induce the expression of HMG-CoA reductase and/or function by inducing oxidative stress. Nevertheless, additional studies are needed to clarify the effect of uremic toxins on the function and/or expression of HMG-CoA reductase.

Statins is always the conversion from the active open acid form into the inactive lactone form and vice versa. SIM is present in equilibrium between acid (active form) and lactone (inactive form) in human blood [28], but PRV is exclusively present in acid (active form) in human serum [29]. Meanwhile, Cerivastatin-induced apoptosis is associated with intracellular acidification on RD cells [30]. Therefore, We think that the conversion from the inactive form to the active form by intracellular acidification can play an important role of cytotoxicity of statins. If statins-induced intracellular acidification is affected by prolonged exposure to uremic toxins, affected regulation mechanism of intracellular pH has effect on the equilibrium between the statin acid and lactone. Nevertheless, additional studies are needed to clarify regulation mechanism of intracellular pH, and also the equilibrium between the statin acid and lactone.

## 3. Experimental Section

### 3.1. Chemicals

PRV, CMPF, and CoQ_10_ were purchased from Cayman Chemical Co., (Ann Arbor, MI, USA). SIM was purchased from Toronto Research Chemicals Inc., (North York, ON, Canada). CDDP, clonazepam and propidium iodide (PI) were obtained from Wako Pure Chemical Industries (Osaka, Japan). Annexin V-FITC was purchased from BioLegend Inc., (San Diego, CA, USA). Hippuric acid, 3-Indoxyl sulfate, mevalonate, FOH, and GGOH were from Sigma-Aldrich Chemical Co., (St. Louis, MO, USA). Indole-3-acetic acid was purchased from Nacalai Tesque, Inc., (Kyoto, Japan). Squalene and lovastatin was obtained from Tokyo Chemical Industry Co., LTD. (Tokyo, Japan). Dolichol was purchased from Avanti Polar Lipids Inc., (Alabaster, AL, USA). The CellQuanti-Blue™ Cell Viability Assay Kit was purchased from BioAssay Systems (Hayward, CA, USA).

### 3.2. Cell Culture

RD cell expresses a number of muscle-specific proteins and has been used as a model in which to study myotoxicity effects of statins [31,32]. The RD cell line was purchased from American Type Culture Collection (Manassas, VA, USA). RD cells were plated at a density of 1 × 10^6^ cells per 10 cm dish in Dulbecco’s modified Eagle’s medium (DMEM, Invitrogen, Grand Island, NY, USA) supplemented with 10% fetal bovine serum (FBS, Thermo Fisher Scientific Inc., Waltham, MA, USA), 100 U/mL penicillin, and 100 µg/mL streptomycin (Nacalai Tesque) (growth medium) at 37 °C with 5% CO_2_ for 3 or 4 days. To induce differentiation, the cells were cultured in DMEM supplemented with 1% FBS and penicillin-streptomycin (differentiation medium) for 7 days. The differentiation medium refreshed on the 3rd or 4th day.

### 3.3. Evaluation of Cytotoxicity

Differentiated RD cells were plated at a density of 5 × 10^3^ cells per well into 96-well plates in differentiation medium containing the following test compounds for 3 days. The final concentrations of the uremic toxins, PRV, SIM, and CDDP (test compounds) were 1–1024 µM, 15.625 nM to 4096 µM, 0.122 nM to 32 μM, and 8 nM to 512 µM, respectively.

Cells were pre-treated with the four uremic toxins at concentrations of 2 or 400 µM CMPF, 180 or 400 µM hippuric acid, 3 or 20 µM indole-3-acetic acid, and 20 or 200 µM 3-indoxyl sulfate. These concentrations reflect the unbound or total serum concentrations in patients with ESRF, respectively [33,34,35]. RD cells were pre-treated with CMPF, hippuric acid, indole-3-acetic acid, 3-indoxyl sulfate, and a combination of all four uremic toxins for the 7-day differentiation period.

In a simultaneous treatment experiment, cells were treated with SIM and mevalonate pathway-related compounds for 3 days as follows: 1000 µM mevalonate, 10, 20, 50, or 100 µM FOH, 10, 20, 50 or 100 µM GGOH, 30 µM squalene, 30 µM CoQ_10_, and 30 µM dolichol. After treatment with the test compounds for 3 days, cell viability was measured using a CellQuanti-Blue™ kit (CellQuanti-Blue™ 10 μL per 100 μL of culture media) and a microplate reader (excitation wavelength = 535 nm, emission wavelength = 590 nm, GENios, Tecan, Seestrasse, Switzerland).

The LC_50_ value was calculated using the non-linear least squares program (MULTI) as follows:

(1) $$L\;=\;L_{\max}\times \;(1-\frac{C^{\text{γ}}}{C^{\text{γ}}+{{LC}_{50}}^{\gamma }})$$

where L, Lmax, C, and γ are cell viability (% of control), maximum cell lethality, drug concentration in the medium, and sigmoid function, respectively. The Control is no toxin, no statin, same incubation time.

In the uptake experiments of PRV and SIM, after removal of differentiation medium, cells pre-treated with 2 µM CMPF, 180 µM hippuric acid, 3 µM indole-3-acetic acid, or 20 µM 3-indoxyl sulfate were washed with HEPES-HBSS (136.9 mM NaCl, 5.4 mM KCl, 1.3 mM CaCl_2_, 0.4 mM MgSO_4_, 0.5 mM MgCl_2_, 0.3 mM NaHPO_4_, 0.4 mM KH_2_PO_4_, 4.2 mM NaHCO_3_, 0.06 mM phenolsulfonphthalein, 5.56 mM glucose, and 25 mM HEPES) and preincubated at 37 °C for 20 min with HEPES-HBSS. Uptake was initiated by applying HEPES-HBSS with 100 µM PRV or 10 µM SIM at 37 °C. After a predetermined time period, uptake was terminated by suctioning HEPES-HBSS with statin and cells were washed with 2 mL of ice-cold phosphate buffered saline (PBS; 136.9 mM NaCl, 2.7 mM KCl, 1.5 mM KH_2_PO_4_ and 8.1 mM Na_2_HPO_4_) and then were suspended in 1.3 mL of ultra pure water. Samples were frozen at −80 °C until the assay. To determine the concentration of PRV and SIM, freezing and thawing cell suspension solution (1 mL) was performed with liquid-liquid extraction methods using methanol (100 µL) with 1 µg/mL clonazepam as an internal standard, 20 mM sodium citrate buffer (pH 5.00, 500 µL), 2-propanol (500 µL) and diethyl ether (4.5 mL) for PRV, or acetonitrile (200 µL) with 2 µg/mL lovastatin as an internal standard, 20 mM sodium citrate buffer (pH 4.50, 500 µL) and acetonitrile (3 mL) for SIM. And then dried-up under N_2_ at 50 °C, suspended in ultra pure water (200 µL) for PRV, or supernatant after centrifugation (1630× *g* for 30min) for SIM. The accumulation of statins assessed using high performance liquid chromatography (HPLC) system. The HPLC (Shimadzu Corp., Kyoto, Japan) consisted of pump (LC-10AS), an UV detection (SPD-6A), and an integrator (C-R4A). The flow rate of mobile phase was 1.0 mL/min, and the column effluent was monitored at 239 nm for PRV, 238 nm for SIM. Separation was achieved using a reversed-phase column (4.0 × 250 mm; Inertsil ODS-3, GL science). The mobile phase was 20 mM sodium citrate buffer (pH 3.00)/acetonitrile [72:28] for PRV, 20 mM sodium citrate buffer (pH 4.50)/acetonitrile [40:60] for SIM. The retention time of PRV, clonazepam, SIM and lovastatin were 21, 35, 22, and 16 min, respectively. Cytomorphology of differentiated RD cells assessed using laser scanning microscopy equipped with a 405 nm diode laser, 10 × ocular and objective lens (LSM510METS Ver. 4.2, Carl Zeiss Co. Ltd., Oberkochen, Germany).

### 3.4. Assessment of Apoptosis

Cells treated with statin (800 µM PRV or 3 µM SIM) for 36 h were suspended at a concentration of 1 × 10^6^ cells/mL in ice-cold buffer (10 mM HEPES, 140 mM NaCl, 2.5 mM CaCl_2_, pH 7.4). Annexin V-FITC (5 μL) and 50 µg/mL PI solution (1 μL) were added to 100 μL cell suspension, and incubated for 15 min in the dark. The reaction solution was diluted five-fold in the same buffer, and then analyzed using FACS Calibur™ (Becton Dickinson, Franklin Lakes, NJ, USA). Apoptotic cells were defined as those that stained annexin V-FITC-positive and PI-negative.

### 3.5. Statistical Analysis

Measured and LC_50_ values were expressed as mean ± standard deviation (S.D.) and median (95% confidence intervals), respectively. The significance of differences between mean values was determined using *post hoc* Tukey-Kramer multiple comparison test with Stat View (SAS Institute Inc., Cary, NC, USA); *p* < 0.05 was considered significant. Non-overlapping confidence intervals were considered statistically significant.

## 4. Conclusions

In conclusion, this study demonstrated that uremic toxins augmented statin-induced apoptosis and cytotoxicity via small G-protein geranylgeranylation. Therefore, the increase in statin-induced rhabdomyolysis in patients with ESRF could be the result of accumulation of uremic toxins. Since the evaluated uremic toxins are protein bound, they are difficult to remove by dialysis. Alternative measures to lower their concentrations, like dietary regimen, could be considered. In addition, to diminish the ADR, the statins with the least additive effect to the cytotoxicity of uremic toxins should be preferred. Another strategy is the use of mevalonate pathway-related compounds, which have no relation to cholesterol synthesis, such as GGPP and FPP. We hope that this information will contribute to more appropriate future use of statins in patients with ESRF.
